# Effects of trees, gardens, and nature trails on heat index and child health: design and methods of the Green Schoolyards Project

**DOI:** 10.1186/s12889-020-10128-2

**Published:** 2021-01-07

**Authors:** Kevin Lanza, Melody Alcazar, Deanna M. Hoelscher, Harold W. Kohl

**Affiliations:** 1 Michael and Susan Dell Center for Healthy Living, School of Public Health in Austin, The University of Texas Health Science Center at Houston, 1616 Guadalupe St. Suite 6.300, Austin, TX 78701 USA; 2 Austin Parks and Recreation Department, 919 W 28th 1/2 St, Austin, TX 78705 USA; 3 Department of Epidemiology, Human Genetics, and Environmental Sciences, School of Public Health in Austin, The University of Texas Health Science Center at Houston, 1616 Guadalupe St. Suite 6.300, Austin, TX 78701 USA; 4 Department of Kinesiology and Health Education, The University of Texas at Austin, 2109 San Jacinto Blvd, Austin, TX 78712 USA

**Keywords:** Health equity, School physical activity, Climate change adaptation, Urban Heat Islands, Temperature, Playgrounds, Joint-use parks, Outdoor nature play

## Abstract

**Background:**

Latinx children in the United States are at high risk for nature-deficit disorder, heat-related illness, and physical inactivity. We developed the Green Schoolyards Project to investigate how green features—trees, gardens, and nature trails—in school parks impact heat index (i.e., air temperature and relative humidity) within parks, and physical activity levels and socioemotional well-being of these children. Herein, we present novel methods for a) observing children’s interaction with green features and b) measuring heat index and children’s behaviors in a natural setting, and a selection of baseline results.

**Methods:**

During two September weeks (high temperature) and one November week (moderate temperature) in 2019, we examined three joint-use elementary school parks in Central Texas, United States, serving predominantly low-income Latinx families. To develop thermal profiles for each park, we installed 10 air temperature/relative humidity sensors per park, selecting sites based on land cover, land use, and even spatial coverage. We measured green features within a geographic information system. In a cross-sectional study, we used an adapted version of System for Observing Play and Recreation in Communities (SOPARC) to assess children’s physical activity levels and interactions with green features. In a cohort study, we equipped 30 3rd and 30 4th grade students per school during recess with accelerometers and Global Positioning System devices, and surveyed these students regarding their connection to nature. Baseline analyses included inverse distance weighting for thermal profiles and summing observed counts of children interacting with trees.

**Results:**

In September 2019, average daily heat index ranged 2.0 °F among park sites, and maximum daily heat index ranged from 103.4 °F (air temperature = 33.8 °C; relative humidity = 55.2%) under tree canopy to 114.1 °F (air temperature = 37.9 °C; relative humidity = 45.2%) on an unshaded playground. 10.8% more girls and 25.4% more boys interacted with trees in September than in November.

**Conclusions:**

We found extreme heat conditions at select sites within parks, and children positioning themselves under trees during periods of high heat index. These methods can be used by public health researchers and practitioners to inform the redesign of greenspaces in the face of climate change and health inequities.

**Supplementary Information:**

The online version contains supplementary material available at 10.1186/s12889-020-10128-2.

## Background

Children in modern times are experiencing “nature-deficit disorder,” described as the human costs of alienation of nature [1], and are consequently missing the benefits of engaging with nature such as a stronger sense of place, improvements in physical and mental health, greater environmental knowledge, and pro-environment attitudes as an adult [2]. In the United States (US), children spend three times as many hours on the computer or watching television as they do playing outdoors [3]. Furthermore, access to nature is an environmental justice issue: individuals who are Latinx, low-income, and/or with low levels of education have less access to vegetation [4, 5].

These same populations lacking access to nature are also at risk for heat-related illnesses, such as heat exhaustion and heat stroke [6]. Communities of color and low-income families disproportionately live in areas characterized by urban heat islands [7], the phenomenon in which cities experience higher temperatures than nearby areas due to high amounts of impervious materials, lack of vegetation, morphology, and waste heat from industrial processes [8].

Along with their high risk for nature-deficit disorder and heat-related illness, Black and Latinx children from low-income families are less physically active than other groups [9, 10]. Fewer than half of all US children are reaching recommended physical activity levels [11]. This lack of physical activity poses a serious public health threat: children who are not sufficiently active are more likely to develop several chronic diseases, such as obesity and type 2 diabetes [12]. To compound the problem, elevated temperatures have been found to be negatively associated with children’s physical activity [13].

With children spending a significant portion of their time at school [14], public health practitioners have promoted child health through green schoolyards—“natural spaces (that) are used as outdoor classrooms to enhance learning outcomes and create daily wellness for the children they serve” [15]. Greenspaces have been found to lower air temperatures (through shading and evapotranspiration from vegetation) and improve human thermal comfort [5, 16]. Schoolyard greening, in particular, has been shown to contribute to children’s physical, mental, and social-emotional well-being [17–20]. However, public health researchers have not fully explored the relations between green schoolyards, temperatures, and child health.

In response, we developed the Green Schoolyards Project to establish whether school parks can serve as a tool for urban heat island adaptation and health promotion in divested communities at risk of disconnect from nature (and its associated health consequences) and heat-related illness. The specific aims of the Green Schoolyards Project are to determine how green features—trees, gardens, and nature trails—in joint-use elementary school parks impact a) heat index within parks; b) physical activity levels of predominantly Latinx children from low-income families; and c) psychosocial and academic outcomes (i.e., social-emotional learning skills, misconduct at school, and standardized test scores) of these children. Herein, we detail the research design and methods of the Green Schoolyards Project, which are novel and innovative in two major ways: a) direct observation of children’s interaction with green features at multiple physical sites per park; and b) time-matching of objective measurements of heat index, children’s geographic location, and children’s physical activity levels in relation to location of green features. We demonstrate the importance of these methods by presenting a selection of baseline results: certain sites within parks were characterized by extreme heat conditions and children positioned themselves under trees during periods of high heat index.

## Methods

### Research design

The Green Schoolyards Project consisted of two separate studies: a) a serial cross-sectional study focused on physical sites within parks, and b) a prospective cohort study focused on individual students. The cross-sectional study was designed to examine the associations between heat index, children’s physical activity, and interaction with green features for multiple sites (e.g., playground, track, and soccer field) per park on multiple times per day. The cohort study followed a selection of students from each school affiliated with these parks to assess the impact of green features—the amount of which differs per park—on student’s physical activity levels during recess for different heat index conditions, along with their connection to nature, social-emotional learning skills, misconduct, and standardized test scores.

### Conceptual model

Research design of the Green Schoolyards Project was based on the social-ecological model and attention restoration theory. The social-ecological model states multiple levels of influence—individual, social, environmental, and policy—impact health behaviors (e.g., physical activity), and these influences interact across levels to impact behavior [21]. Effective health interventions focus on behavior-specific influences, and intervene at multiple levels of influence. In general, attention restoration theory proposes exposure to natural environments replenishes the ability to concentrate, a cognitive resource that can be depleted [22, 23]. Natural settings are considered restorative because these environments provide a) respite from everyday stressors, b) space that feels extensive, c) objects that fascinate and require little concentration, and d) intrinsic compatibility with humans [23].

**Fig. 1:**
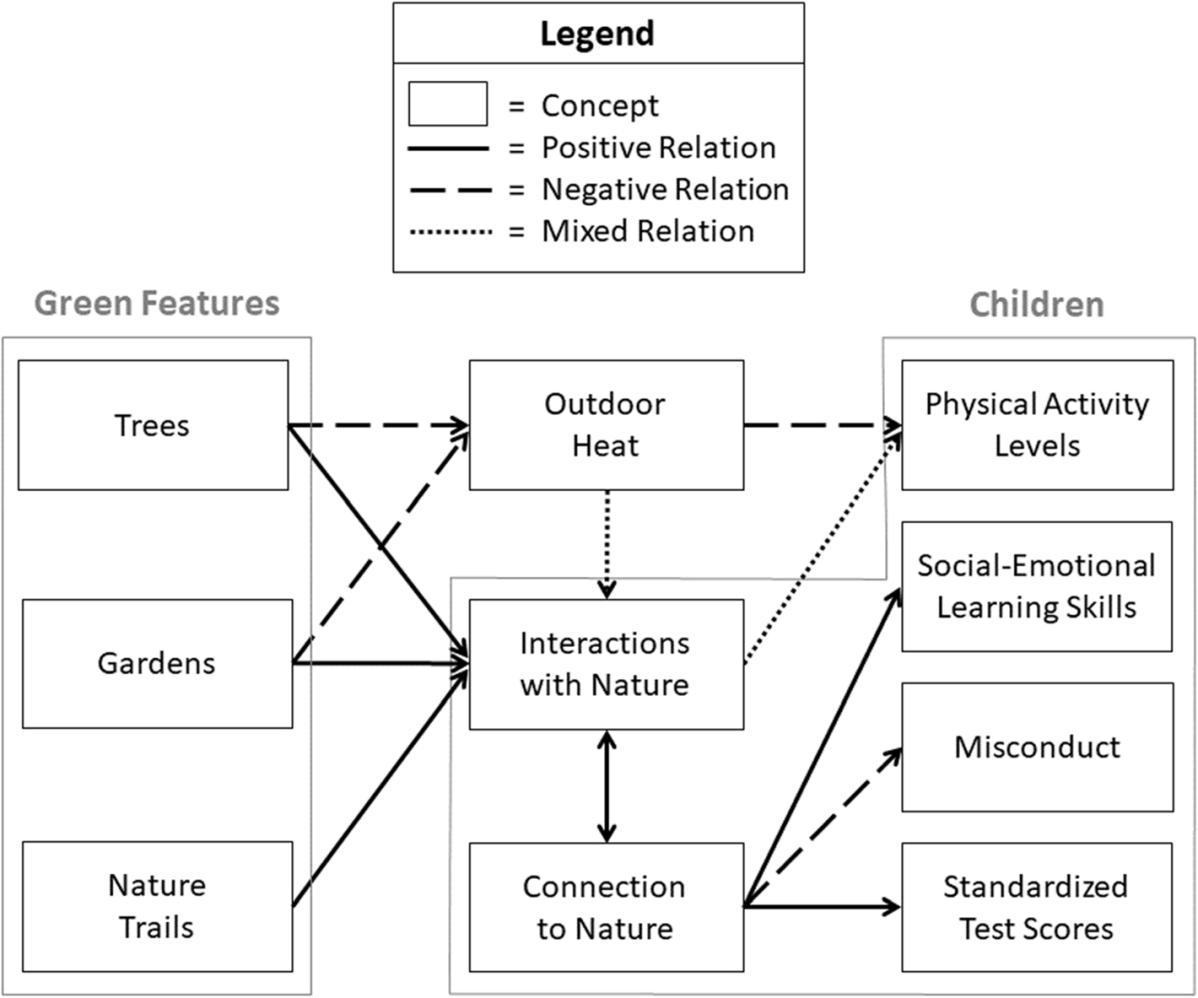
Conceptual model of Green Schoolyards Project

From these theoretical underpinnings, we developed a conceptual model of the Green Schoolyards Project that illustrates the concepts of interest and relations between these concepts (Fig. 1). The model provides our logic for testing green features in school parks as a strategy for improving health, psychosocial, and academic outcomes of children. We posited that trees and gardens will decrease outdoor heat and that nature trails, which surround gardens but are not directly vegetated, will not significantly impact outdoor heat. When subjected to outdoor heat, children will engage in less physical activity due to thermal discomfort. Trees, gardens, and nature trails will inherently provide more opportunities for children to physically interact with nature, yet outdoor heat will have a mixed impact on these physical interactions: children will seek natural settings providing protection from outdoor heat (e.g., park sites under tree shade) while not seeking and potentially avoiding natural settings without heat protection (e.g., unshaded park sites). The relation between children’s physical interactions with nature and their physical activity levels will depend on the interaction with nature (e.g., climbing trees versus resting under tree shade) and whether the setting is intended for physical activity (e.g., playground under tree canopy versus picnic tables under tree canopy). Lastly, children’s physical interactions with nature will offer opportunities for beneficial outcomes, such as mentally connecting with nature and restoring ability to concentrate. Improved concentration will increase children’s social-emotional learning skills and standardized tests scores, and decrease their misconduct at school.

### Project sites and green features

Three elementary school parks within a school district in Central Texas, US, were used for this study. The project was a comparative analysis between similar parks with different levels of green features. Three schools met our initial selection criteria: serving populations greater than 85% economically disadvantaged Latinx; located in ZIP codes with low Nature Factor scores; joint-use agreements between the school district and the city Parks and Recreation Department permitting the surrounding community to use parks after school hours; and equivalent park features (e.g., playgrounds, soccer fields, running tracks, and basketball courts).

The selection criterion of Nature Factor is defined as the sum of Nature Factor Ratings of all parks within a ZIP code [24]. Nature Factor Rating is the sum of four park-level ratings: park acreage rating, Trust for the Public Land land use rating, National Recreation and Parks Association park status rating, and tree canopy rating [25]. High values for Nature Factor Ratings (e.g., high park acreage, designed lands, open park status, and high levels of tree canopy) correspond to high Nature Factor scores (i.e., higher levels of nature present in that ZIP code). The three selected schools are in ZIP codes with Nature Factor scores of 121, 118, and 198, respectively, which are relatively low compared to those of other ZIP codes (*n* = 53; min. = 0; max. = 712; mean = 150; standard deviation = 143).

The three school parks were characterized by different profiles of green features: the “intervention park” had added green features (i.e., trees, wildflower meadow, and nature trail); the “low-green park” had relatively low amounts of historical green features (i.e., trees); and the “high-green park” had relatively high amounts of historical green features (i.e., trees, wildlife habitat garden, and nature trail).

Landscape vegetation—including trees and gardens—was planted in each park to provide visual enhancement; learning, play, and recreation opportunities; and a calming and welcoming environment. The school district is responsible for maintaining trees, and individual schools and their communities are responsible for maintaining gardens and nature trails. The wildflower meadow at the intervention park is a 1383m^2^ pollinator-supporting grassland consisting of dense, tall wildflowers and grasses native to Texas [26]. The meadow includes signage and a weaving, 20 m-long, 1.5 m-wide, nature trail—a secondary or tertiary path that branches from a primary track, trail, or pathway and composed of materials such as decomposed granite, compacted dirt, and stepping stones. To encourage use of the meadow, a nature themed-story in English- and Spanish-language was installed on temporary signage placed along the trail. The wildlife habitat garden at the high-green park is a 247m^2^ dense cover of native grasses and shrubs that serves as a haven for local and migratory species by providing food, cover, and places to raise young [27]. This garden includes a nature trail that is nearly identical to the trail in the wildflower meadow at the intervention park. For the Green Schoolyards Project, both wildflower meadow and wildlife habitat garden are categorized as gardens because of similar characteristics. The low-green park had no gardens or nature trails.

For each school park, we calculated tree canopy cover using i-Tree Canopy, a publicly available tool that uses random point sampling to estimate the percentage of tree canopy cover for a predefined area [28]. Although trees at the intervention park were more abundant and evenly distributed than trees at parks at the other schools, tree canopy cover was only 8.5% (standard error = 1.97) because trees planted were saplings. The low-green park had 11.5% (standard error = 2.26) tree canopy cover, with most trees clustered in the far northwest corner. The high-green park had 22.5% (standard error = 2.95) tree canopy cover, with relatively large trees on the park’s periphery.

### Project period

We designed data collection to take place on 18 days over the fall semester in 2019, which will be duplicated in 2020 for a total of 36 study days. Each year, study days consist of two September weeks (i.e., five weekdays and one weekend day per week) and one November week (i.e., five weekdays and one weekend day). We selected September and November because these months have historically high and moderate temperature conditions, respectively: the weather station at the city’s major airport recorded monthly mean average air temperatures of 26.4 °C in September and 15.3 °C in November from 2009 to 2018 [29]. Prior to undertaking any project activities, we received approval of project protocols by the institutional review board at The University of Texas Health Science Center at Houston (HSC-SPH-19-0502) and the school district. We also received informed consent from participants’ parents and written assent from study participants.

### Measurement of heat index

We measured heat index—the combination term for air temperature and relative humidity that captures what the temperature feels like [30]—by semi-permanently installing 10 HOBO MX2302A external air temperature/relative humidity sensor data loggers (Onset Computer Corporation, MA) at each park. Previous studies have used comparable networks of in situ sensors to monitor microclimatic conditions of a given area [31–33]. Measurement of near-surface air temperatures is advantageous over the use of land surface temperatures as a proxy for air temperatures, as research has shown land surface temperatures are not directly comparable to air temperatures [34, 35]. In situ measurement of air temperatures has been found to be more useful for estimating short-term, actual temperature exposures than using land surface temperatures or the percentage of impervious surface [36]. Designed for outdoor use, the MX2302A model collects air temperature (±0.2 °C from 0 to 70 °C) and relative humidity data (±2.5% from 10 to 90%), and is configured to wirelessly link with the free HOBOmobile app on a cell phone or tablet [37], permitting efficient collection of air temperature and relative humidity data by project members.

**Fig. 2:**
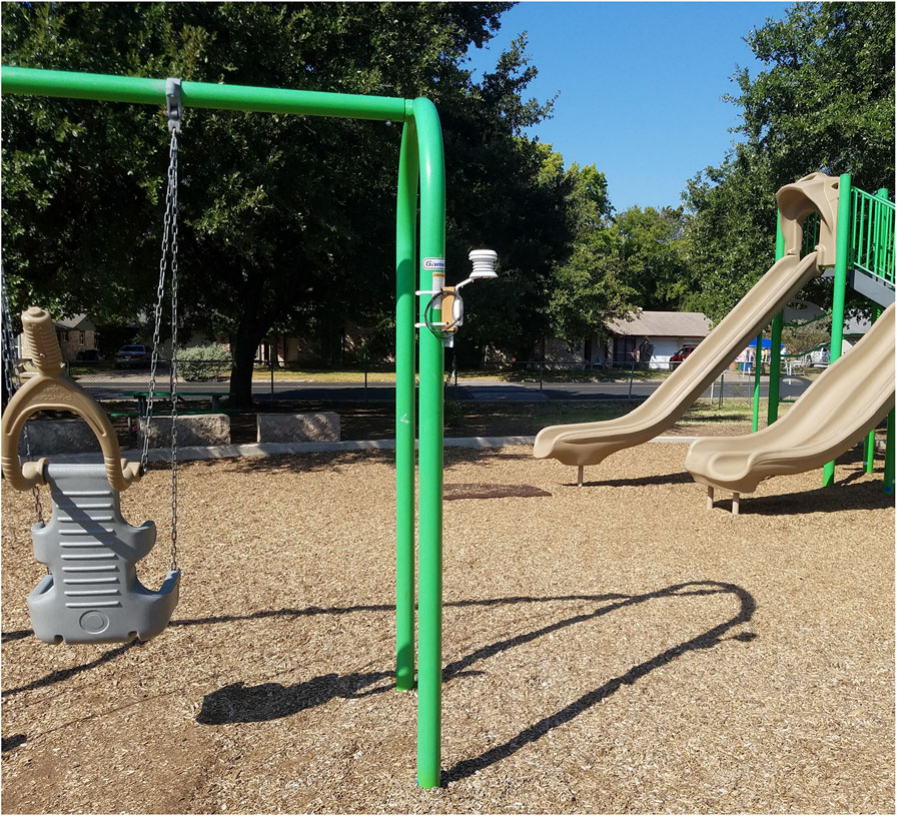
HOBO MX2302A external air temperature/relative humidity sensor data logger on swing set at low-green park

Before deploying HOBO sensors, we encased each sensor in an RS3-B solar radiation shield (Onset Computer Corporation, MA), which results in improved temperature measurement accuracy by protecting the sensor from absorption of incoming solar radiation and resultant heat gain. In another attempt to improve temperature measurement accuracy, we attached the radiation shield (encasing the external sensor) and data logger to a 2 × 2” piece of weather-treated lumber, which serves as a physical buffer minimizing heat transfer between the sensor and the installation surface in the park, such as a metal swing set pole (Fig. 2). We programmed the sensors to record air temperature and relative humidity every 5 min, consistent with previous studies [33, 38, 39].

**Fig. 3:**
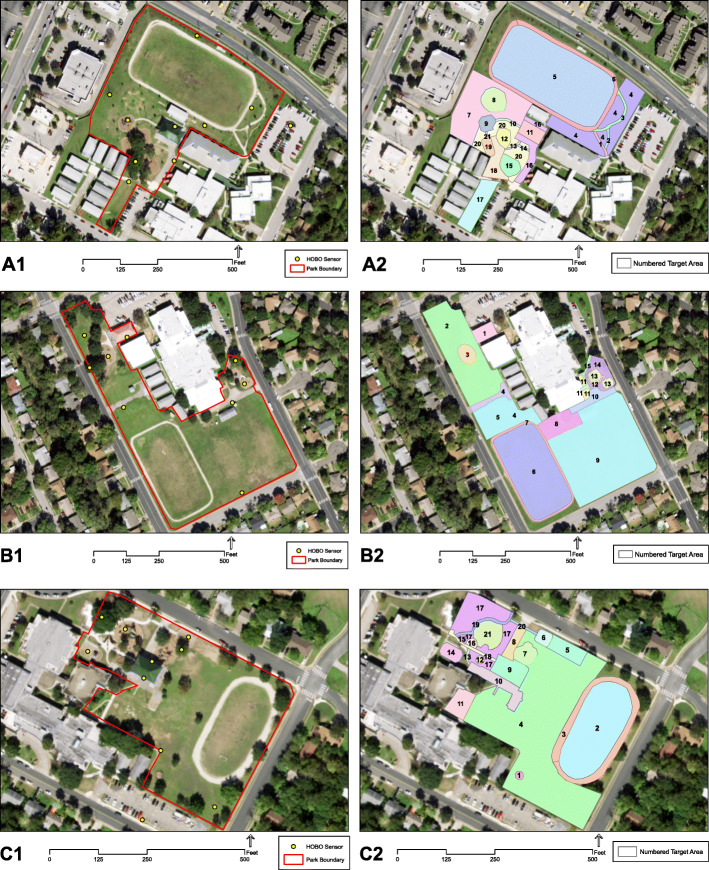
HOBO sensors and SOPARC target areas at (**A1**–**A2**) intervention, (**B1**–**B2**) low-green, and (**C1**–**C2**) high-green parks

For each park, we selected 10 sites for HOBO sensors based on land cover (e.g., grass, pavement, and mulch); land use (e.g., soccer field, basketball court, and playground); comparability across parks; and even spatial coverage (Fig. 3). To include a highly impervious area for comparison within park sites, we installed one of the 10 sensors at each park’s parking lot, just outside park boundaries. To capture air temperature and relative humidity experienced by humans, we installed sensors at two meters above ground level, similar to previous studies [32, 33, 38]. To promote community awareness of our project and deter vandalism, we attached a small laminated tag with a description of the sensor and our contact information to each sensor. Sensors were installed the day before a study week, and removed at the end of each study week.

### Measurement of green features

We identified the location, type, and quantity of green features using four-band, 60 cm orthoimagery taken in November 2018 by the US Department of Agriculture’s National Agriculture Imagery Program [40]. Within a geographic information system (ArcGIS 10.6.1, ESRI, Redlands, CA, USA), we digitized polygons of trees, gardens, and nature trails, an established technique deemed appropriate for the relatively small park areas (i.e., intervention = 21,448 m^2^; low-green = 27,923 m^2^; high-green = 16,187 m^2^) [41].

### Direct observation of parks

The cross-sectional study utilized the System for Observing Play and Recreation in Communities (SOPARC), a validated direct observation tool for assessing the conditions and users of park sites [42]. Following SOPARC protocol, we divided each park into target areas intended for physical activity, such as basketball courts and soccer fields (Fig. 3). On study days during school (i.e., 7:00 and 12:00) and after school (i.e., 16:00 and 18:00), study staff—in pairs for interrater reliability—administered SOPARC by walking from target area to target area and recording what they observed. We adapted SOPARC to measure physical activity levels of children aged 1–12 years old and these children’s interactions with green features. Although previous research has used direct observation to examine the influence of nature on children’s play [43], no research employs SOPARC to quantify the number of children’s interactions with different green features at multiple park sites.

On study days for each target area, trained staff used SOPARC to record the date and time of observation and target area conditions (i.e., whether area was accessible, usable, equipped, supervised, dark, empty, and organized); then, if applicable, to record the primary physical activity (e.g., playing basketball) of female and male children; scan for the physical activity levels of female and male children; and lastly scan for the number of female and male children interacting with green features (i.e., no interaction, under tree canopy or touching tree, interacting with garden, and on nature trail). To employ SOPARC, staff used the iSOPARC application on an electronic tablet for scan counts and input data on a data collection form (see Additional file 1).

### Cohort study sample

For the cohort study, we recruited 40 3rd and 40 4th grade students per school over 2 years, to achieve a final sample size of 30 students per grade, after accounting for attrition. From mid-August 2019 through early September 2019, we recruited participants by convenience sampling. Participant incentives were a total of $35 US dollars/year worth of supermarket gift cards (i.e., $10 for each September study week and $15 for each November study week).

### Measurement of geographic location and physical activity

On study days during recess (i.e., 30-min period of unstructured play under teacher supervision), the cohort sample wore elastic belts around their waist equipped with a Qstarz BT-Q1000XT Global Positioning System (GPS) device (Qstarz Intl Co., Taipei, Taiwan) and an Actigraph wGT3X-BT accelerometer (ActiGraph LLC, FL) to measure geographic location and physical activity levels, over time [44, 45]. We set sampling rates of 15 s for GPS devices and accelerometers [46, 47]. For the separate recess periods per grade, belt distribution began 5 min before recess start, and belt collection occurred once teachers signaled recess end.

### Time-matching of geographic location, physical activity, and heat index

Data from GPS devices, accelerometers, and HOBO sensors will be time-matched, allowing us to know a student’s location, student’s physical activity intensity level, and heat index at that location at 15-s intervals throughout recess. The location of green features will be joined to the time-matched device data, within GIS. Although previous studies have matched children’s geographic location and physical activity levels over time [44–47], this study enables assessment of a child’s experienced heat index and physical activity level at any particular location.

### Measurement of connection to nature, psychosocial and academic outcomes, and school policies

For baseline data collection in November 2019, we collected data on the cohort sample and school policies from three sources. First, we administered aloud a written survey—in both English and Spanish language—to the cohort sample, asking them about their connection to nature using two adapted instruments: Inclusion of Nature with Self and Connection to Nature Index (see Additional file 2) [48–50]. Second, the school district provided individual data for each student on sociodemographic characteristics, social-emotional learning skills (from a student climate survey), number of disciplinary actions for misconduct, and standardized test scores. Lastly, we distributed an annual survey—adapted from a previous study [51]—to ask school principals about policies impacting park access, greening at school parks, and student physical activity.

### Statistical analysis of baseline data

For the selection of baseline findings shared within, we performed analysis over several steps. To develop thermal profiles for each park, we first calculated heat index—from air temperature and relative humidity data recorded by HOBO sensors—using validated equations utilized by US National Weather Service [52, 53]. Heat index is measured in degrees Fahrenheit [54]. Within GIS, we used inverse distance weighting to create spatially continuous thermal profiles for each park. A common interpolation method in urban heat island measurement [55], inverse distance weighting permits estimation of unsampled heat index values between HOBO sensors by averaging sampled heat index values from sensors surrounding each prediction location. We used SOPARC data to understand how children interact with trees during time periods with different temperature conditions, summing observed counts of children under tree canopy or touching trees by sex of child, park, and study period.

## Results

### Thermal profiles of school parks

**Fig. 4:**
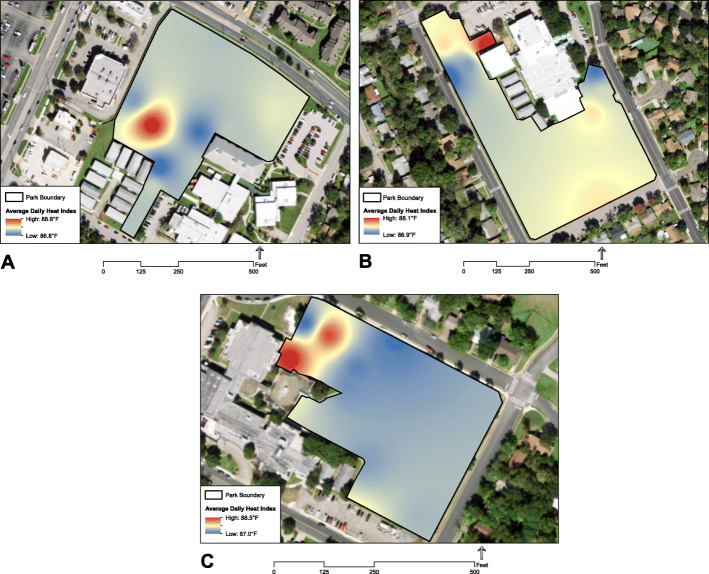
Thermal profiles for (**a**) intervention, (**b**) low-green, and (**c**) high-green parks, September 16–30th 2019

Average daily heat index was 87.4 °F in September and 62.4 °F in November across the three parks, with average daily heat index ranging from 86.8 °F to 88.8 °F from September 16–30th, in 2019 (Fig. 4). The minimum and maximum values of this range originated from the intervention park at two sites less than 50 m apart: a playground under heavy tree canopy (Target Area 18) and an unshaded playground (Target Area 9), respectively (Fig. 3A2).

In September 2019 at the intervention park (Fig. 4a), the canopied playground reached a maximum heat index of 104.3 °F (air temperature = 34.6 °C; relative humidity = 51.0%), whereas the unshaded playground reached 114.1 °F (air temperature = 37.9 °C; relative humidity = 45.2%). At the low-green park (Fig. 4b), the lowest maximum heat index was 103.4 °F (air temperature = 33.8 °C; relative humidity = 55.2%) under heavy tree canopy (Target Area 14), and the highest was 106.9 °F (air temperature = 35.8 °C; relative humidity = 47.4%) at an unshaded playground (Target Area 13). At the high-green park (Fig. 4c), the lowest maximum heat index was 104.2 °F (air temperature = 33.8 °C; relative humidity = 55.8%) at a basketball court under an artificial shade structure (Target Area 9), and the highest was 109.4 °F (air temperature = 35.9 °C; relative humidity = 50.5%) at an unshaded playground (Target Area 14).

### Children’s interaction with green features

When performing SOPARC scans for the number of children interacting with green features, we observed a total of 1229 children in target areas with trees, three children in target areas with gardens, and zero children in target areas with nature trails, during 12 days in September and 6 days in November 2019. In target areas with trees (Table 1), children observed at the low-green park constituted 67.1% of all children observed in September and 65.4% of all children observed in November. Target areas with trees were frequented by a slightly larger percentage of female children than male children in both September (52.4% female) and November (51.8% female). Across the three parks, these target areas had 10.8% more female children and 25.4% more male children under tree canopy or touching trees in September than in November 2019. However, a lower percentage of female and male children at the high-green park interacted with trees in September than in November. Across the three parks, we observed no children interacting with gardens or on nature trails during study days in September and November 2019.

**Table 1:** Children’s interaction with trees at school parks on study days using SOPARC (September, November 2019)

School Park	Study Period (2019)	Children Under Tree Canopy or Touching Trees^**a**^		Total Children Observed^**a**^	
		Female (%)	Male (%)	Female (#)	Male (#)
Intervention	September	59.7	65.7	72	67
	November	50.0	53.7	30	41
Low-Green	September	71.4	77.6	273	228
	November	59.8	44.7	184	132
High-Green	September	19.6	13.3	46	60
	November	63.2	64.5	36	60
Total	September	63.2	64.5	391	355
	November	52.4	39.1	250	233

^a^Observations are for target areas with trees present within its boundaries

## Discussion

### Methodology for climate and health solutions

We provided baseline findings from the Green Schoolyards Project as evidence for the utility of the methods in understanding how green features can moderate place-based climate change and health inequities affecting children. Public health researchers and practitioners can use these methods as a model for exploring how joint-use parks with green features can serve as climate and health solutions for divested communities, in the wake of current climate change and health inequities impacting cities [7]. Understanding how green features in school parks impact heat index and child health is essential due to projected increases in a) urban temperatures from population-driven development; b) global temperatures from greenhouse gas emissions; and c) the intensity, frequency, and duration of extreme heat events from climate change [56].

From thermal profiles of each park (Fig. 4), we found a two-degree range in average daily heat index (86.8–88.8 °F) in September 2019 across the three parks, which is a change detectable by humans [57]. Park sites with heavy tree canopy and artificial shade structures exhibited the lowest heat index values, and unshaded playgrounds exhibited the highest heat index values. These results corroborate those of a previous study that examined the temperatures of surface materials in parks in Phoenix, Arizona, in which researchers found tree canopy and artificial shade structures were associated with significant reductions in surface temperatures, and playground structures exhibited the highest surface temperatures [58]. In September at the intervention park, we found a 9.8 °F difference in maximum heat index between a canopied playground and an unshaded playground—the difference between “Extreme Caution” and “Danger” levels for likelihood of extreme heat disorders with prolonged exposure or strenuous activity, as defined by the US National Weather Service [59].

From using SOPARC to understand children’s interaction with green features, we found more children were interacting with trees in September than in November across the three parks (Table 1). This may suggest children were actively seeking trees—a proven heat management strategy [60, 61]—during high heat index for thermal comfort. Yet this finding was reversed when examining the high-green park independently, which may be related to children’s preference for a playground characterized by sparse canopy cover (i.e., 53.8% of all children observed), and their unwillingness to travel to large trees on the park’s periphery during high heat index. Our finding that no children interacted with gardens or used nature trails may be due to these features being located far from play elements (e.g., slides, ladders, and swings), which have been shown to be associated with more users and more moderate-to-vigorous physical activity [62]. In addition, installation of green features in a park may not induce the use of these spaces: researchers have found that living near sidewalk improvements was not associated with accelerometer-derived physical activity [63]. Lastly, the gardens at the parks (i.e., wildflower meadow and wildlife habitat garden) may not have been aesthetically pleasing enough or functional for children, unlike vegetable and fruit gardens. Children have been found to engage in more physical activity during outdoor, garden-based lessons than during indoor, classroom-based lessons [64]. Seasonally, wildflower meadows may be more attractive to children when flowers and pollinators are present.

### Strengths and limitations

The methods presented herein are unique for adapting a direct observation tool to measure children’s interaction with green features, and for actively documenting heat index and children’s behaviors in a natural setting (i.e., recess period during a typical school day). Other strengths included the use of validated tools for the objective measurement of heat index (i.e., HOBO sensors), conditions and users of park sites (i.e., SOPARC), children’s physical activity levels (i.e., accelerometers), and children’s geographic location (i.e., GPS devices).

One limitation of the Green Schoolyards Project was the uneven spatial distribution of HOBO sensors at each park, which could have reduced the accuracy of air temperature and relative humidity values in the thermal profile for each park. In inverse distance weighting interpolation, an uneven spatial distribution of observational data points results in less accurate predictions between observational data points [65]. We found that achieving an even spatial coverage of sites for HOBO sensors at each park was difficult in practice. Installation at certain sites—in particular soccer fields—would obstruct use of those sites and/or sites lacked a surface for us to attach a sensor at two-meter height. Additional reasons for uneven spatial coverage included the potential for sensor damage, vandalism, and theft at each prospective site. Future studies can develop more accurate thermal profiles by installing HOBO sensors with more even spatial coverage, supplementing the data recorded by installed HOBO sensors with periodic handheld measurements between installation sites, and/or modeling air temperatures from a combination of air temperature and land surface temperature inputs [66].

We experienced two limitations with the use of SOPARC. For observations occurring on study days during school (i.e., 7:00 and 12:00), the number of children present at each park was directly linked to the schedule of the school and teachers, which resulted in significant differences in the number of children observed during school hours across school parks. If a recess period happened to coincide with an observation period, then more children were observed than if there was no overlap between observation and recess period. Each school had different schedules for recess across classes and grades (i.e., pre-kindergarten through 5th grade), and recess periods were sometimes shared among multiple classes and grades. Teachers were in control of the length of recess, and the start and end times of recess often fluctuated. As a solution, researchers can work with school staff to understand the recess schedule, and assign SOPARC observation periods accordingly.

A second limitation of SOPARC was that we did not observe children’s physical activity and interaction with green features simultaneously. Because these two behaviors were observed on different scans, the female and male children observed during the scans for physical activity were not necessarily the same children observed during the scans for interaction with green features. In future work, we will test the use of a single scan to measure both physical activity and interaction with green features, to understand the physical activity levels of children while interacting with green features. Behavior mapping may be preferred over SOPARC because this direct observation method identifies individuals’ specific locations within a study area [67], which allows for the production of design-sensitive results.

Lastly, the cohort sample may be prone to response bias—specifically acquiescence bias and social desirability bias—when responding to survey items about their connection to nature, potentially affecting data quality [68–71].

## Conclusions

We designed the Green Schoolyards Project to determine the relations between green features and heat index at joint-use school parks and the health of children who are at high risk for nature-deficit disorder, heat-related illness, and physical inactivity. From baseline results, we found extreme heat index conditions at school parks, significant differences in heat index across park sites, and more children interacting with trees during periods of high heat index than periods of moderate heat index. The methods presented herein can be adopted by projects in other cities that are exploring how to redesign urban greenspaces to adjust to high heat conditions and eliminate health inequities facing communities. City officials can use findings from implementing these methods to inform the funding of green feature enhancements at parks in areas characterized by urban heat islands and poor health outcomes. In future analyses of the Green Schoolyards Project, we will further examine how trees, gardens, and nature trails at school parks impact heat index within parks and physical activity levels of predominantly Latinx children from low-income families; and how these children’s connection to nature relates to their social-emotional learning skills, misconduct, and standardized test scores. If we find these green features to decrease heat index within parks, increase children’s physical activity levels, and/or exhibit positive associations with children’s psychosocial and academic outcomes, then we can recommend future installments of green features in school parks for child health in a warming world.

## Supplementary Information

**Additional file 1.** System of Play and Recreation in Communities (SOPARC) data collection form. Paper-based form used by study staff to input System of Play and Recreation in Communities (SOPARC) data, adapted to measure physical activity levels of children aged 1–12 years old and these children’s interactions with green features.

**Additional file 2.** Connection to nature survey: Inclusion of Nature with Self and Connection to Nature Index. Connection to nature survey instrument administered annually to cohort sample, based on two instruments: Inclusion of Nature with Self and Connection to Nature Index.

## Data Availability

The datasets used and analyzed during the current study are available from the corresponding author on reasonable request.

## Abbreviations

GIS: Geographic Information System
GPS: Global Positioning System
HOBO: Not an abbreviation/accronym
SOPARC: System for Observing Play and Recreation in Communities
US: United States
