# Supplementary material for: Effects of trees, gardens, and nature trails on heat index and child health: design and methods of the Green Schoolyards Project
Source: BMC Public Health. 2021 Jan 7;21:98. doi: 10.1186/s12889-020-10128-2 (PMC7792068; doi:10.1186/s12889-020-10128-2)
Supplement: Supplementary file 1 — Additional file 1. System of Play and Recreation in Communities (SOPARC) data collection form. Paper-based form used by study staff to input System of Play and Recreation in Communities (SOPARC) data, adapted to measure physical activity levels of children aged 1–12 years old and these children’s interactions with green features. [file 12889_2020_10128_MOESM1_ESM.pdf]

Date: Park: Observer: Period (circle one): Morning Lunch Afternoon Evening

Target Area _____________
Target Area #

Start Time _________

Conditions of Target Area

Accessible (e.g., not locked or rented to others) ☐ Yes ☐ No
Usable (e.g., is not excessively wet or windy) ☐ Yes ☐ No
Equipped (e.g., removable balls available) ☐ Yes ☐ No
Supervised (e.g., presence of teachers) ☐ Yes ☐ No
Dark (e.g., insufficiently lit) ☐ Yes ☐ No
Empty (e.g., scan area is empty) ☐ Yes ☐ No
Organized (i.e., team sporting event) ☐ Yes ☐ No

Comments:

PEOPLE	ACTIVITY	ACTIVITY LEVEL			INTERACTION WITH GREEN FEATURES			
**Children**	**Primary Activity**	**S**	**W**	**V**	**No Interaction with Green Features**	**Under/Touching Tree**	**Interacting with Garden**	**On Nature Trail**
Female								
Male								
