# Supplementary material for: Effects of trees, gardens, and nature trails on heat index and child health: design and methods of the Green Schoolyards Project
Source: BMC Public Health. 2021 Jan 7;21:98. doi: 10.1186/s12889-020-10128-2 (PMC7792068; doi:10.1186/s12889-020-10128-2)
Supplement: Supplementary file 2 — Additional file 2. Connection to nature survey: Inclusion of Nature with Self and Connection to Nature Index. Connection to nature survey instrument administered annually to cohort sample, based on two instruments: Inclusion of Nature with Self and Connection to Nature Index. [file 12889_2020_10128_MOESM2_ESM.docx]

Unique Participant ID:

1. Please circle the picture below that best describes your relationship with the natural environment (self = you; nature = the environment):


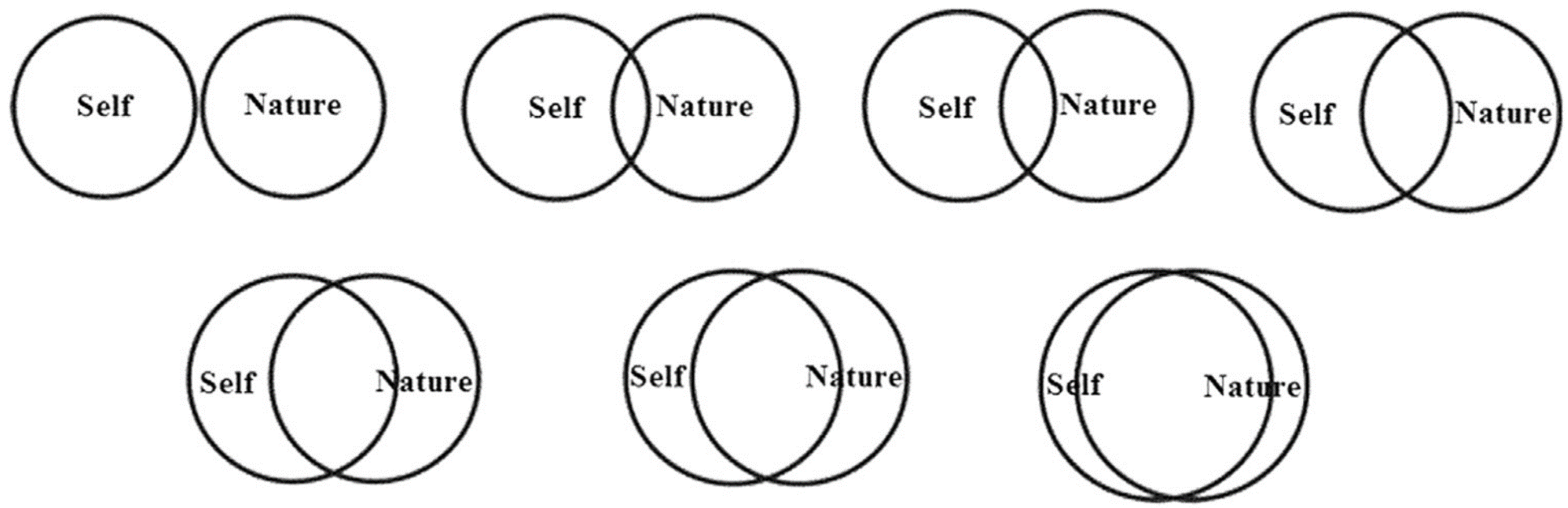


1. Please circle the face below that best describes how much you agree or disagree with each of the following statements:

*(Circle only one face per statement. If you need to change your answer, erase completely).*

**I like to hear different sounds in nature**


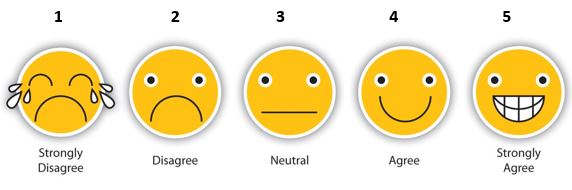


**I like to see wildflowers in nature**


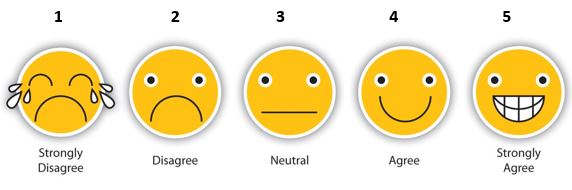


**When I feel sad, I like to go outside and enjoy nature**


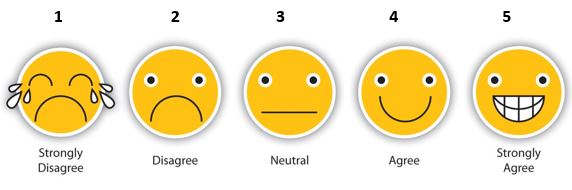


**Being in the natural environment makes me feel peaceful**


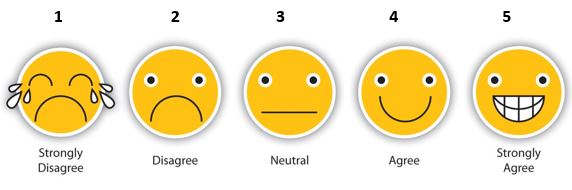


**I like to garden**


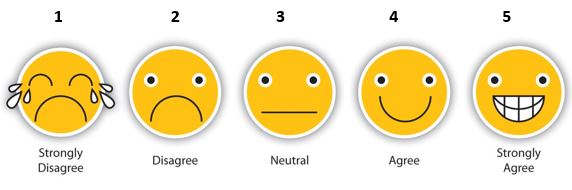


**Collecting rocks and shells is fun**


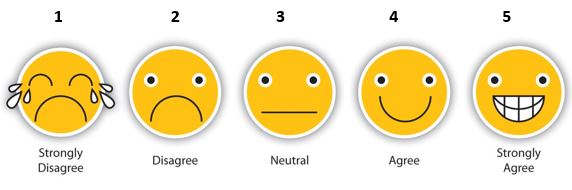


**I feel sad when wild animals are hurt**


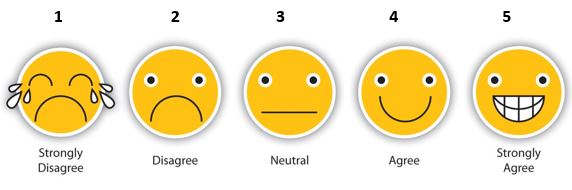


**I like to see wild animals living in a clean environment**


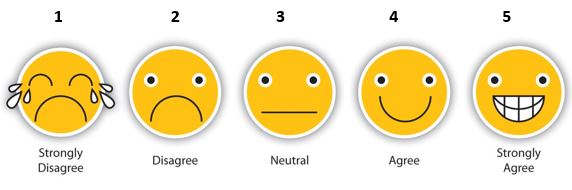


**I enjoy touching animals and plants**


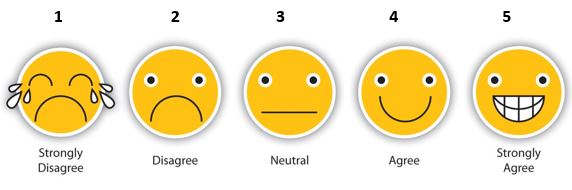


**Taking care of animals is important to me**


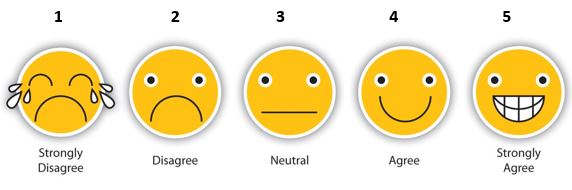


**Humans are part of the natural world**


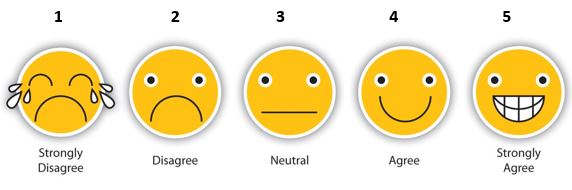


**People cannot live without plants and animals**


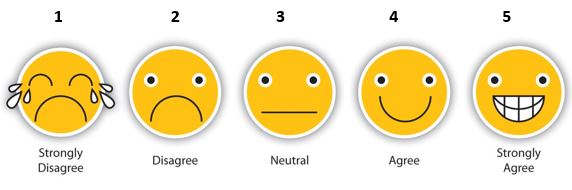


**Being outdoors makes me happy**


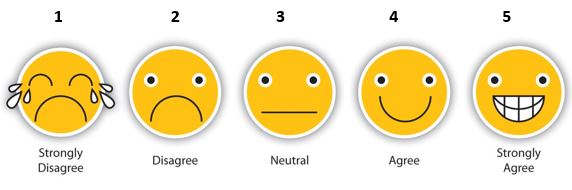


**My actions will make the natural world different**


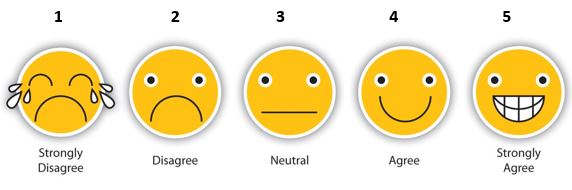


**Picking up trash on the ground can help the environment**


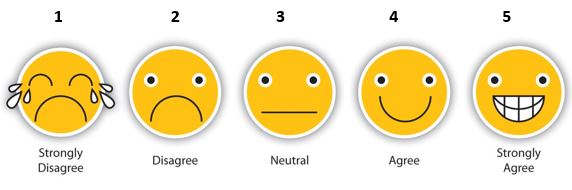


**People do not have the right to change the natural environment**


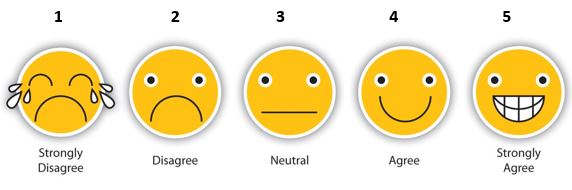


***Thank you for your participation!***
